# Healthcare Mistreatment, State-Level Policy Protections, and Healthcare Avoidance Among Gender Minority People

**DOI:** 10.1007/s13178-022-00748-1

**Published:** 2022-07-15

**Authors:** Kristen D. Clark, Sean Luong, Mitchell R. Lunn, Elena Flowers, Esmaeil Bahalkeh, Micah E. Lubensky, Matthew R. Capriotti, Juno Obedin-Maliver, Annesa Flentje

**Affiliations:** 1 Department of Nursing, University of New Hampshire, Durham, NH USA; 2 Department of Community Health Systems, School of Nursing, University of California San Francisco, San Francisco, CA USA; 3 The PRIDE Study/PRIDEnet, Stanford University School of Medicine, Stanford, CA USA; 4 Division of Nephrology, Department of Medicine, Stanford University School of Medicine, Stanford, CA USA; 5 Department of Epidemiology and Population Health, Stanford University School of Medicine, Stanford, CA USA; 6 Department of Physiological Nursing, School of Nursing, University of California San Francisco, San Francisco, CA USA; 7 Department of Health Management & Policy, University of New Hampshire, Durham, NH USA; 8 Department of Psychology, San Jose State University, San Jose, CA USA; 9 Department of Obstetrics and Gynecology, Stanford University School of Medicine, Stanford, CA USA; 10 Department of Psychiatry, School of Medicine, Alliance Health Project, University of California San Francisco, San Francisco, CA USA

**Keywords:** Health policy, LGBTQ, Gender minority, Healthcare access, Social determinants of health

## Abstract

**Introduction:**

This study examined whether past experiences of mistreatment in healthcare were associated with greater healthcare avoidance due to anticipated mistreatment among gender minority (GM) people. We evaluated whether state-level healthcare policy protections moderated this relationship.

**Methods:**

Data from the 2018 Annual Questionnaire of The PRIDE Study, a national longitudinal study on sexual and gender minority people’s health, were used in these analyses. Logistic regression modeling tested relationships between lifetime healthcare mistreatment due to gender identity or expression and past-year healthcare avoidance due to anticipated mistreatment among GM participants. Interactions between lifetime healthcare mistreatment and state-level healthcare policy protections and their relationship with past-year healthcare avoidance were tested.

**Results:**

Participants reporting any lifetime healthcare mistreatment had greater odds of past-year healthcare avoidance due to anticipated mistreatment among gender expansive people (*n* = 1290, OR = 4.71 [CI]: 3.57–6.20), transfeminine people (*n* = 263, OR = 10.32 [CI]: 4.72–22.59), and transmasculine people (*n* = 471, OR = 3.90 [CI]: 2.50–6.13). Presence of state-level healthcare policy protections did not moderate this relationship in any study groups.

**Conclusions:**

For GM people, reporting lifetime healthcare mistreatment was associated with healthcare avoidance due to anticipated mistreatment. State-level healthcare policy protections were not a moderating factor in this relationship. Efforts to evaluate the implementation and enforcement of state-level policies are needed. Continued efforts to understand instances of and to diminish healthcare mistreatment of GM people are recommended.

**Supplementary Information:**

The online version contains supplementary material available at 10.1007/s13178-022-00748-1.

## Introduction

GM people (individuals whose gender identity is not aligned with that traditionally associated with the sex that was assigned to them at birth) experience considerable stigma related to their gender identity. Stigma may be enacted through discrimination and mistreatment, which is a cause of health inequalities (Hatzenbuehler et al., 2013; Testa et al., 2017). Stigma can be understood as a contributor to health inequalities described within the minority stress model (Meyer, 2003; Testa et al., 2017). The minority stress model, originally developed to describe the experiences of cisgender sexual minority men (Meyer, 1995), describes the additional stress GM people may be exposed to, above and beyond the typical day-to-day stress among the general population, and poses a source of chronic stress (Testa et al., 2017). This additional stress related to one’s marginalized gender identity occurs in distal and proximal forms. Distal stressors may occur as prejudice-related or stigmatizing events or experiences (Meyer, 2003; Testa et al., 2017). Proximal stressors are the negative self-view of one’s GM identity or internalization of harmful societal views of their identity, the expectation or anticipation of mistreatment or harm, and/or the act of concealing one’s GM identity. However, stigma may also occur structurally, as harmful policies or exclusion from resources (King et al., 2020; Link & Phelan, 2001; White Hughto et al., 2015).

Discrimination and other types of mistreatment experienced by GM people are pervasive within healthcare settings (Ayhan et al., 2019; Cicero et al., 2019). A national study of GM adults found that 33% of GM respondents reported experiencing mistreatment when interacting with healthcare providers (James et al., 2016). Healthcare mistreatment encompasses a wide range of experiences including inadequate healthcare provider knowledge about GM health needs, refusal of healthcare services, verbal harassment, and physical violence (Ayhan et al., 2019; Cicero et al., 2019; Lambda Legal, 2010). Healthcare mistreatment has a profound effect on health-seeking behavior (Ben et al., 2017; Bindman et al., 2021; Moscoso-Porras & Alvarado, 2018). One national study found that 23% of GM respondents reported that they did not seek care due to fear of disrespect or mistreatment related to their gender identity (James et al., 2016). However, until more recently, studies on GM healthcare access and experiences have not been inclusive of diverse gender identities in their approach (Cicero et al., 2019).

Differences among gender identity groups are important to evaluate because we know that gender-expansive (e.g., nonbinary, genderqueer) people, for example, are less likely to access clinics and providers where their practice is focused on transgender people whose gender identity is binary (i.e., solely feminine or masculine; Feldman et al., 2021**)**. This could result in a greater likelihood of encountering less affirming healthcare settings, such as emergency departments or other acute care environments (Hughto et al., 2018; Mafham et al., 2020; Stotzer et al., 2013). Even when healthcare settings are aimed at providing gender-affirming care, gender-expansive people report an overemphasis on the transition toward a gender binary (Lykens et al., 2018). In some cases, they report greater occurrences of being misgendered than GM people with a binary gender identity (Goldberg et al., 2019). Gender expansive people have also reported greater avoidance of routine checkups and having no usual source of care when compared to transgender women (Gonzales & Henning-Smith, 2017).

Other gender identity groups are important to evaluate in research on healthcare access and experiences as well. Transgender men have reported greater difficulty accessing gender-affirming care and HIV services (Bockting et al., 2013) as well as a lower likelihood of obtaining preventative health screenings when compared to transgender women (Hoy-Ellis et al., 2022). These differences in healthcare avoidance across gender identity groups are an important area of focus as avoidance of preventative healthcare or treatment of chronic health conditions is a contributor to health inequalities. Harmful outcomes of healthcare avoidance observed among GM people include poor general health (Seelman et al., 2017), depression, suicidal ideation, suicide attempts, and seeking gender-affirming treatments (e.g., hormones) from sources outside of the healthcare system (Glick et al., 2018a, b; Xavier et al., 2007).

While the relationship between healthcare mistreatment and healthcare avoidance has been explored, the role of state-level discrimination protections in this relationship is less understood. Few previous studies have evaluated the relationship between state-level healthcare discrimination protections and the experiences of GM people. GM people living in states with more protective state healthcare policies, such as laws prohibiting discrimination on the basis of gender or an absence of laws offering exceptions for healthcare refusal based on religious reasons, had lower odds of avoiding healthcare (Goldenberg et al., 2020a), greater mental health services utilization (Goldenberg et al., 2020b), and had seen a provider more recently (Du Bois et al., 2018) compared to GM people living in states with few healthcare protections. However, no studies examined whether the presence of state-level policy protections acts as a moderator, reducing the rate of healthcare avoidance among GM people who have previously experienced healthcare mistreatment.

The purpose of this study was to assess the relationship between lifetime healthcare mistreatment due to gender identity or expression with reported past-year avoidance of healthcare services among individual subgroups of GM people (i.e., gender expansive people, transfeminine people, and transmasculine people). We hypothesized that reports of lifetime healthcare mistreatment due to gender identity or expression were associated with greater past-year healthcare avoidance due to anticipated mistreatment among GM people. Secondly, we sought to determine whether residing in a state with healthcare policy protections moderated the relationship between lifetime reports of healthcare mistreatment and past-year healthcare avoidance due to anticipated mistreatment. We hypothesized that GM people who lived in a state with protective healthcare policies and reported previous healthcare mistreatment were less likely to avoid healthcare than GM people who reside in a state with neutral or harmful healthcare policies.

## Methods

### Study Sample

This study analyzed data collected from the 2018 Annual Questionnaire of The Population Research in Identity and Disparities for Equality (PRIDE) Study. The PRIDE Study is an online, national, longitudinal cohort study on the health of sexual and gender minority people. Recruitment for The PRIDE Study took place through PRIDEnet (a network of LGBTQ + -focused community partners) members, online communications (e.g., blog posts, newsletters, social media advertising), in-person outreach at conferences and events with the distribution of The PRIDE Study promotional items, and word-of-mouth (Lunn et al., 2019a, b). The PRIDE Study participants are required to be 18 years of age or older, reside in the USA or its territories, and be able to read and understand English (Lunn et al., 2019a, b). Participants included in our analyses selected a gender identity that was different than the sex that they were assigned at birth, selected more than one gender identity, or selected a gender-expansive gender identity such as genderqueer or nonbinary (*n* = 2574) and completed responses to survey questions about healthcare mistreatment and healthcare avoidance. The Institutional Review Board at Stanford University and the University of California San Francisco as well as the Research Advisory Committee and Participant Advisory Committee for The PRIDE Study approved this study.

### Measures

#### Demographics

Demographic characteristics assessed were age, race/ethnicity, sexual orientation, gender identity, sex assigned at birth, education level, household gross income, state of residence, and insurance status. Age was calculated by subtraction of participants’ birth date from the date that the survey was completed. Race and ethnicity were measured in a manner that allowed participants to select all options that apply: American Indian or Alaska Native, Asian, Black, Hispanic/Latino/Spanish, Middle Eastern or North African, Native Hawaiian or other Pacific Islander, White, and “none of these fully describe me” with a free text response box. Sexual orientation was measured by asking participants “What is your current sexual orientation.” Participants could select all options that apply: asexual, bisexual, gay, lesbian, pansexual, queer, questioning, same-gender loving, straight/heterosexual, and “another sexual orientation” with a free text response box. Gender identity was measured by asking participants “what is your current gender identity?” Participants could select all options that apply: genderqueer, man, transgender man, transgender woman, woman, and “another gender identity” with a free text response box. Sex assigned at birth was measured by asking, “What was your sex assigned at birth, for example on your original birth certificate?” Participants had the option to respond either female or male. Education level was measured with 10 options ranging from “no schooling” to “professional degree.” This was coded in our analyses as a 4-level variable: “no high school diploma,” “high school/GED graduate or some college,” “college degree (2- or 4-year),” and “graduate degree.” Individual income was measured with 11 options ranging from $0 to $100,000 or more, which were used in the analysis in these increments. Participants’ ZIP code was used to identify their state of residence and matched with healthcare policy protection data.

#### Lifetime Healthcare Mistreatment and Denial of Care

Participants were asked the following “yes”/”no” questions regarding healthcare mistreatment: (i) “Have you ever been denied or given lower quality medical care?” and (ii) “Have you ever been denied or given lower quality mental health care?” Participants who responded “yes” to either were asked, “Was any of this discrimination in a medical setting due to your…” and could select all that apply: ability/disability status, age, body size/weight/shape, gender expression, gender identity, race/ethnicity, sexual orientation, or “something else.” Participants who indicated they experienced healthcare mistreatment and/or denial of care and that the reason was related to gender expression or gender identity were coded as yes (1). Participants who indicated they had not experienced healthcare mistreatment and/or denial of care or who indicate yes but the reason was not related to gender expression or gender identity were coded as no (0). This coding process was applied separately to each of the two healthcare mistreatment variables (medical care and mental healthcare settings).

#### Past-Year Healthcare Avoidance Due to Anticipated Mistreatment

Participants were asked “Was there a time in the past year when you needed to see a healthcare provider but did not because you thought you would be disrespected or mistreated?” Those who answered “yes” were considered to have avoided healthcare due to anticipated mistreatment.

#### State-Level Healthcare Policy Protections

The Movement Advancement Project (MAP) is a nonprofit organization that provides structural-level data collection about existing policies and laws relating to sexual orientation or gender identity (Movement Advancement Project, 2018). Positive points are assigned to policies that are protective for sexual and gender minority people and negative points are assigned to policies that are harmful. Tallies related to healthcare policy were applied in our analyses. State-level healthcare policy tallies were determined by totaling points from four types of policies related to health and safety pertaining specifically to GM people:

(i) “private health insurance nondiscrimination laws” (0 or 1 point)-this policy prohibits private insurance plans from denial or exclusion from coverage due to their gender identity. The year that this policy was implemented by the state is provided in Supplementary Table 1.
(ii) “health insurance providers banned from excluding coverage from transgender-specific care” (0 or 1 point)-this policy prevents insurers from explicitly excluding coverage for procedures or treatment related to gender-affirming interventions.
(iii) “state Medicaid policies related to coverage for transgender people” (− 1 to 1 point)-states that explicitly cover medically necessary gender-affirming interventions as part of Medicaid are scored as 1, states that do not explicitly cover but also do not explicitly exclude are scored as 0, states with explicit exclusions related to procedures or treatment related to gender-affirming interventions are scored as − 1.
(iv) “transgender-inclusive health benefits for state employees” (0 or 0.5 point)-this policy indicates whether health insurance plans for state employees explicitly cover medically necessary gender-affirming interventions.
(v) “state criminalizes exposure to and/or transmission of HIV” (− 0.5 or 0)-this policy indicates that transmission of HIV, or perceived transmission, is a criminal act, which is largely viewed as counterproductive to public health efforts (Galletly & Pinkerton, 2006).

State-level policy protections have a total possible range of − 1.5 to 3.5 points. States were categorized as having a harmful level or neutral healthcare policies (≤ 0 points) or a protective level of healthcare policies (> 0 points). Data were extracted for state-level healthcare policies as of January 1, 2017, so that policies were in effect one year prior to data collection in our sample.

### Statistical Analysis

Using the participant-reported gender identity and sex assigned at birth variables, participants were assigned to one of three gender groups (Flentje et al., 2020). The gender-expansive group was comprised of people who selected a nonbinary gender identity (e.g., genderqueer, genderfluid), selected both feminine and masculine gender identities (e.g., selecting both transgender man and woman) or neither feminine nor masculine gender identities (e.g., agender) and assigned any sex at birth. The transfeminine group was comprised of people who selected a feminine gender identity such as woman, transwoman, or demigirl and assigned male sex at birth. The transmasculine group was comprised of people who selected a masculine gender identity such as man, transman, or demiboy and assigned female sex at birth. Participants who did not complete survey questions about healthcare mistreatment and healthcare avoidance or who did not report their ZIP code were dropped from the final analysis (*n* = 553).

Descriptive statistics were used to describe demographics and the frequencies of healthcare discrimination and healthcare avoidance. Sexual orientation variables were individually dichotomized to form indicator variables (e.g., gay coded as yes/no or 0/1, American Indian or Alaskan Native coded as yes/no or 0/1). Race/ethnicity variables were recoded following the same procedure.

For the first model, logistic regression models identified associations between lifetime healthcare mistreatment due to gender identity or expression and past-year healthcare avoidance due to anticipated mistreatment. For the second model, logistic regression models were used to evaluate the relationship between reported lifetime healthcare mistreatment and the presence of state-level healthcare policy protections and included interaction terms. Interaction terms evaluated whether the presence of state-level healthcare policy protections moderated the relationship between lifetime healthcare mistreatment and healthcare avoidance due to anticipated mistreatment.

Each logistic regression model was then run in the total sample and then separately for each of the three gender groups: gender expansive people, transfeminine people, and transmasculine people All models covaried age, sexual orientation, race/ethnicity, household income, education level, and whether the state had accepted Medicaid expansion. The Medicaid expansion (part of the Affordable Care Act in 2014) offered states the option to expand eligibility for Medicaid services and is covaried within models because it may have increased accessibility of healthcare services in states where it was accepted (year of implementation by the state is provided in Supplementary Table 1; Kaiser Family Foundation, 2020; Patient Protection & Affordable Care Act, 2010).

In order to identify if the relationship between lifetime healthcare mistreatment and past year healthcare avoidance remained, even when accounting for similar types of policies between states, a k-means cluster analysis was performed to cluster the states by the types of policy scores present. The cluster analysis clustered states based on the proximity of the 5 policy items ([i] “private health insurance non-discrimination laws,” [ii] “health insurance providers banned from excluding coverage from transgender-specific care,” [iii] “state Medicaid policies related to coverage for transgender people,” [iv] “transgender-inclusive health benefits for state employees,” and [v] “state criminalizes exposure to and/or transmission of HIV”) to one another and form the most similar clusters in terms of these policy items (McLachlan, 1992).

Before implementing k-means clustering, policy items were scaled to ensure their homogeneity. A Euclidean distance matrix was computed using the scaled data. A dendrogram to determine the appropriate number of clusters (i.e., k) and k-means clustering were then implemented using the resulted distance matrix. A logistic regression model was run in each of the clusters separately, covarying age, sexual orientation, race/ethnicity, household income, education level, and whether the state had accepted Medicaid expansion. A Bonferroni correction was applied, setting the significance level at *p* < 0.0045. Analyses were run using Stata version 15 (Stata Corp, 2017).

## Results

### Sample Characteristics

A total of 2024 participants across all three gender groups (*n* = 1290 gender expansive people*, n* = 263 transfeminine people, *n* = 471 transmasculine people) were included in our analyses. The median age for the total sample was 28.02 years. Table 1 contains a detailed description of the sample. Nearly 35% of participants had completed high school, 39% completed a 2- or 4-year college degree, and 25% had a graduate degree. Almost 15% of participants reported a household income of less than $20,000 per year, while 44% reported a household income of over $60,000 per year. Approximately 33% of participants reported having experienced healthcare mistreatment due to gender identity and/or expression during their lifetime. Over 33% of participants reported avoiding healthcare due to anticipated mistreatment (Table 1). Approximately 45% of participants were living in a state with harmful or neutral state healthcare policy tallies (≤ 0 points).

**Table 1:** Characteristics of gender minority participants from the PRIDE Study 2018 Annual Questionnaire (*n* = 2024)

**Variable**	**Total sample**	**Gender Expansive**	**Transfeminine**	**Transmasculine**
*n* (%)	2024	1290 (63.74)	263 (12.99)	471 (23.27)
*Personal characteristics*				
Age, interquartile range, Median	23.20–35.42, 28.02	22.86–3.03, 27.05	27.95–53.62, 38.16	22.87–35.18, 27.67
Race/ethnicity (*n*, %)^a^				
American Indian or Alaskan native	78 (3.85)	55 (4.26)	7 (2.66)	16 (3.40)
Asian	109 (5.39)	81 (6.28)	7 (2.66)	21 (4.46)
Black, African American, or African	72 (3.56)	47 (3.64)	5 (1.90)	20 (4.25)
Hispanic, Latino, or Spanish	103 (5.09)	63 (4.88)	11 (4.18)	29 (6.16)
Middle Eastern or North African	31 (1.53)	25 (1.94)	2 (0.76)	4 (0.85)
Native Hawaiian or Pacific Islander	12 (0.59)	10 (0.78)	1 (0.38)	1 (0.21)
White	1866 (92.19)	1187 (92.02)	244 (92.78)	435 (92.36)
Another race/ethnicity	47 (2.32)	34 (2.64)	7 (2.66)	6 (1.27)
Sexual orientation (*n*, %)^a^				
Asexual	353 (17.44)	275 (21.32)	31 (11.79)	47 (9.98)
Bisexual	626 (30.93)	428 (33.18)	65 (24.71)	133 (28.24)
Gay	368 (18.18)	218 (16.90)	15 (5.70	135 (28.66)
Lesbian	339 (16.75)	215 (16.67)	123 (46.77)	1 (0.21)
Pansexual	500 (24.70)	340 (26.36)	65 (24.71)	95 (20.17)
Queer	1188 (58.70)	914 (70.85)	54 (20.53)	220 (46.71)
Questioning	81 (4.00)	44 (3.41)	20 (7.60)	17 (3.61)
Same-gender loving	146 (7.21)	106 (8.22)	16 (6.08)	24 (5.10)
Straight/heterosexual	92 (4.55)	9 (0.70)	18 (6.84)	65 (13.80)
Another sexual orientation	141 (6.97)	119 (9.22)	12 (4.56)	10 (2.12)
*Socioeconomic characteristics*				
Education level				
No high school degree	24(1.19)	13 (1.01)	2 (0.76)	9 (1.91)
High school/GED graduate or some college	694 (34.32)	429 (33.31)	90 (34.22)	175 (37.15)
College degree, 2- or 4-year	793 (39.22)	511 (39.67)	113 (42.97)	169 (35.88)
Graduate degree	511 (25.27)	335 (26.01)	58 (22.05)	118 (25.05)
Household income				
< $20,000	288 (14.57)	200 (15.91)	28 (10.89)	60 (12.99)
$20,000–$39,999	458 (23.18)	300 (23.87)	62 (24.12)	96 (20.78)
$40,000–$59,999	359 (18.17)	230 (18.30)	34 (13.23)	95 (20.56)
$60,000 +	871 (44.08)	527 (41.93)	133 (51.75)	211 (45.67)
Experienced lifetime healthcare mistreatment	676 (33.40)	390 (30.23)	83 (31.56)	203 (43.10)
Live in a state with protective state-level healthcare policies	1102 (54.45)	726 (56.28)	143 (54.37)	233 (49.47)
Avoided healthcare due to fear of mistreatment within the past year	684 (33.79)	435 (33.72)	62 (23.57)	187 (39.70)

^a^Variables were not mutually exclusive; therefore, totals may add up to over 100%

### Lifetime Healthcare Mistreatment and Past-Year Healthcare Avoidance

We evaluated the relationship between reported lifetime healthcare mistreatment and healthcare avoidance during the past year in logistic regression models (primary results in Table 2; odds ratios, confidence intervals, and *p* values for covariates are reported in Supplementary Table 2). Our first models found that those who reported healthcare mistreatment during their lifetime had greater odds of avoiding healthcare during the past year due to anticipated mistreatment than those that had not reported healthcare mistreatment in the total sample (OR = 4.47, 95% CI: 3.61–5.63; *p* < 0*.*001) and in all three gender groups: gender expansive people (OR = 4.71, 95% CI 3.57–6.20; *p* < 0*.*001), transfeminine people (OR = 10.32; 95% CI 4.72–22.59; *p* < 0*.*001), and transmasculine people (OR = 3.90; 95% CI 2.50–6.13; *p* < 0*.*001). Age and sexual orientation were also associated with avoiding healthcare during the past year due to anticipated mistreatment (*p* < 0.0045; Supplementary Table 2).

**Table 2:** Associations between lifetime healthcare mistreatment and healthcare avoidance due to anticipated mistreatment in transfeminine, transmasculine, and gender-expansive groups

**Models**	**Total sample**			**Gender expansive**			**Transfeminine**			**Transmasculine**		
Model 1	OR	95% CI	*p*	OR	95% CI	*p*	OR	95% CI	*p*	OR	95% CI	*p*
Experienced lifetime healthcare discrimination	**4.47**	**3.61, 5.63**	***p* < 0.001**	**4.71**	**3.57, 6.20**	***p* < 0.001**	**10.32**	**4.72, 22.59**	***p* < 0.001**	**3.90**	**2.50, 6.13**	***p* < 0.001**
Model 2												
Experienced lifetime healthcare discrimination (direct effect)	**3.82**	**2.80, 5.23**	***p* < .001**	**4.52**	**2.96, 6.92**	***p* < 0.001**	4.03	1.35, 12.04	*p* = 0.013	**3.21**	**1.75, 5.88**	***p* < 0.001**
State-level healthcare policy protections (direct effect)	0.85	0.65, 1.11	*p* = 0.223	0.96	0.69, 1.34	*p* = 0.807	0.45	0.15, 1.35	*p* = 0.156	0.57	0.31, 1.06	*p* = 0.074
Experienced lifetime healthcare discrimination *x* state-level healthcare policy protections	1.33	0.88, 2.02	*p* = 0.177	1.07	0.62, 1.86	*p* = 0.801	5.02	1.11, 22.76	*p* = 0.037	1.55	0.66, 3.65	*p* = 0.313

Bolded values indicate significance at the < 0.0045 level

OR, odds ratio; CI, confidence interval

Covariates in analyses included age, sexual orientation, race/ethnicity, household income, and education level

### State-Level Healthcare Policy Protections as a Moderator

Model 2 examined whether state-level healthcare policy protections moderated the relationship between lifetime healthcare mistreatment due to gender identity or expression and past-year healthcare avoidance due to anticipated mistreatment (Table 2). No statistically significant relationship was found in the interactions between healthcare policy protections and lifetime healthcare mistreatment among the total sample or any of the gender groups.

### Cluster Analysis of Lifetime Healthcare Mistreatment and Past-Year Healthcare Avoidance

**Fig. 1:**
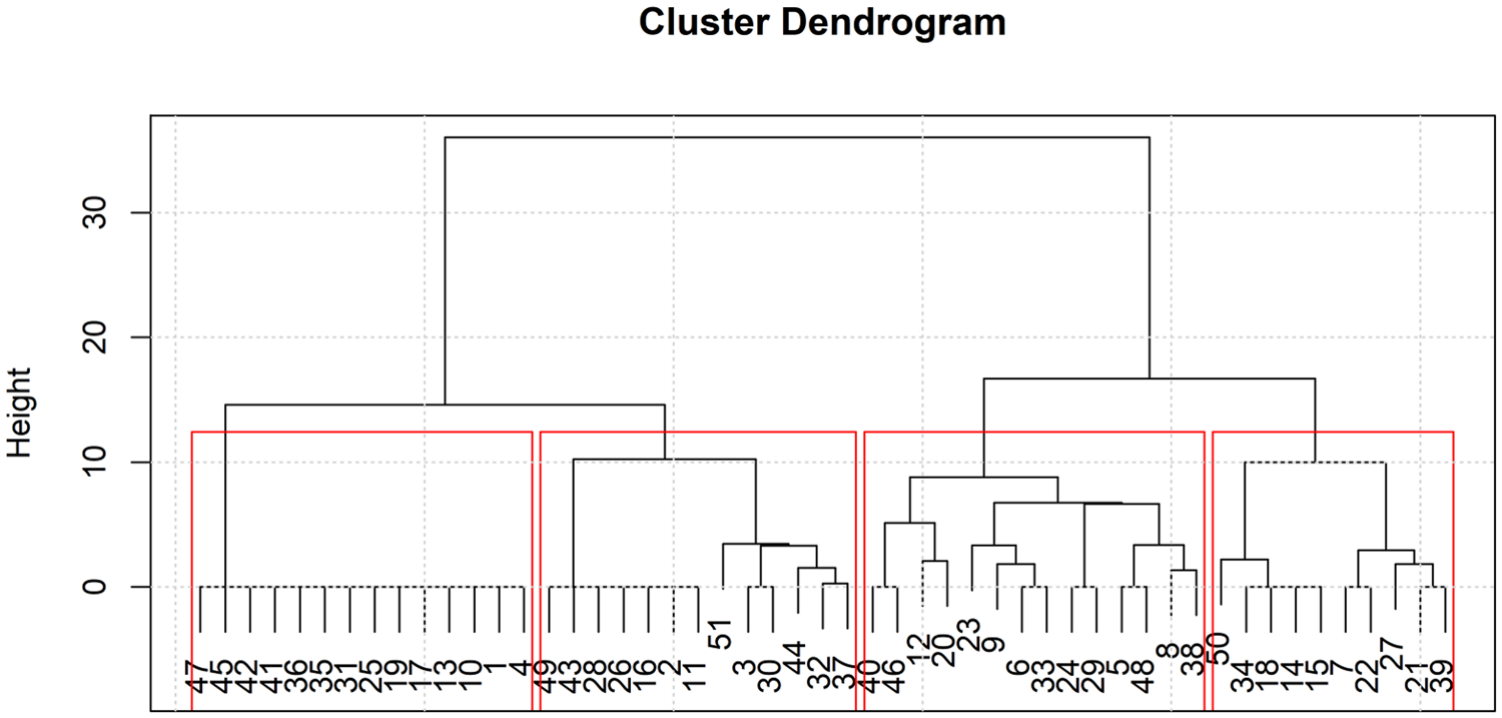
A dendrogram of the state cluster results

**Fig. 2:**
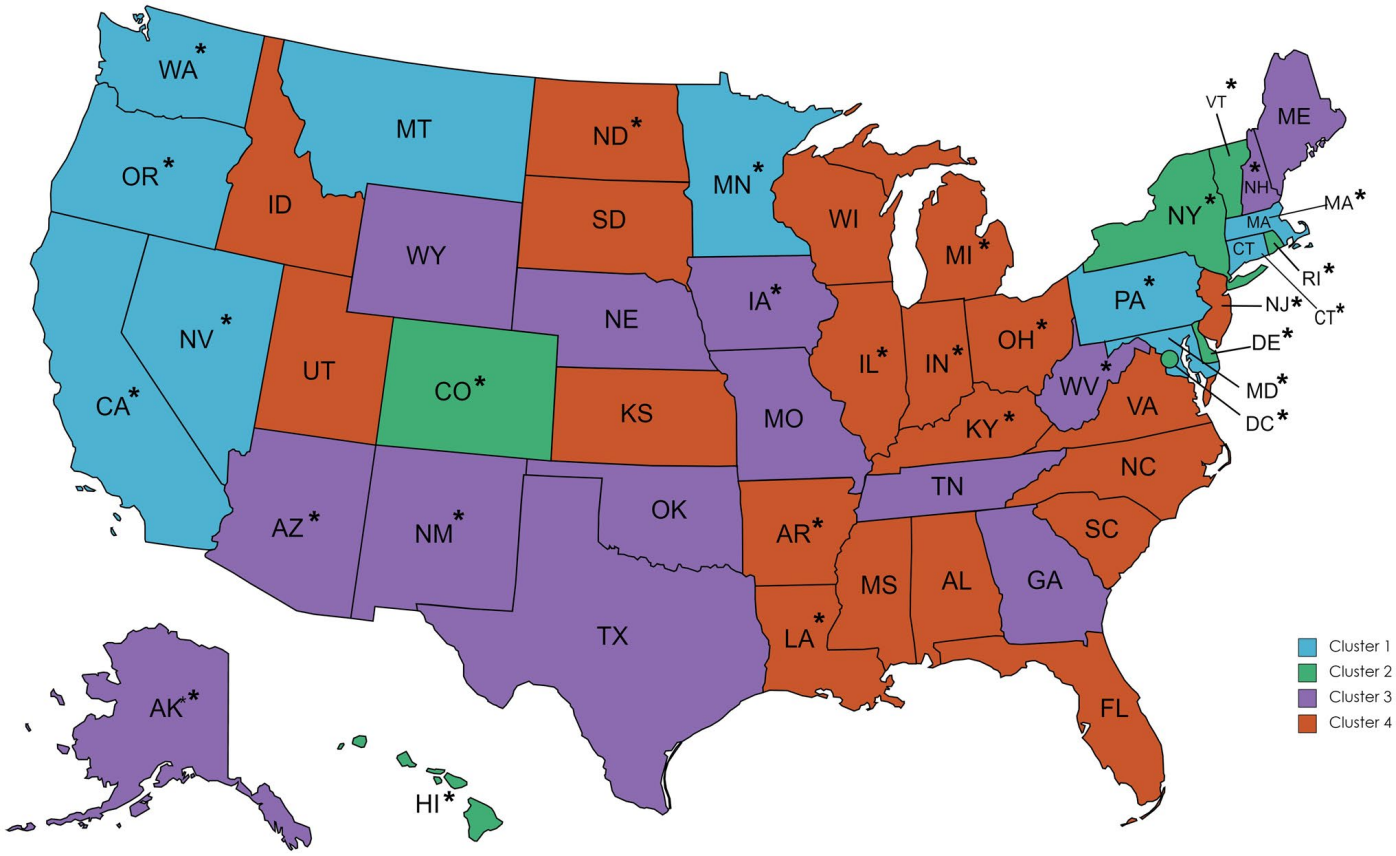
A map of the USA color coded by the 4 clusters and identification of states with Medicaid expansion (*)

**Fig. 3:**
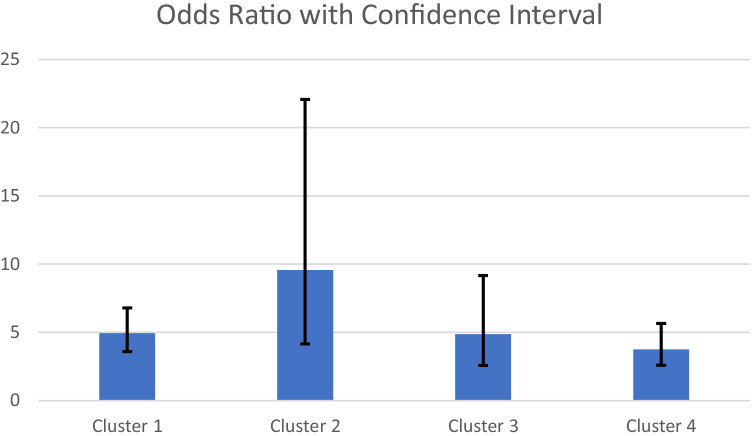
Visualization of odds ratios with confidence intervals

A dendrogram of the cluster results is provided in Fig. 1. A map with the states grouped by cluster is available in Fig. 2. A solution of 4 clusters of states was chosen for the post hoc cluster analysis, where cluster 1 was the states with the most protective healthcare policies and descending to cluster 4 where the states were grouped as those with the least protective healthcare policies. The characteristics of the samples as grouped by the identified clusters are reported in Supplementary Table 3. We evaluated the relationship between reported lifetime healthcare mistreatment and healthcare avoidance during the past year in logistic regression models among the 4 identified state clusters (Table 3). Our models showed that those who reported healthcare mistreatment during their lifetime had greater odds of avoiding healthcare during the past year due to anticipated mistreatment than those that had not reported healthcare mistreatment in all four clusters: cluster 1—OR = 4.94, 95% CI: 3.59–6.80; *p* < 0*.*001; cluster 2—OR = 9.57, 95% CI 4.15–22.07; *p* < 0*.*001; cluster 3—OR = 4.86; 95% CI 2.58–9.17; *p* < 0*.*001; cluster 4—OR = 3.75; 95% CI 2.59–5.65; *p* < 0*.*001 (Fig. 3). The confidence intervals suggest that the relationship between healthcare avoidance and past-year healthcare mistreatment remained consistent even among those living in states with similar policies.

**Table 3:** Associations between lifetime healthcare mistreatment and healthcare avoidance due to anticipated mistreatment grouped by state clusters and covarying state Medicaid expansion

**Model**	**Cluster 1**			**Cluster 2**			**Cluster 3**			**Cluster 4**		
	OR	95% CI	*p*	OR	95% CI	*p*	OR	95% CI	*p*	OR	95% CI	*p*
Experienced lifetime healthcare discrimination	**4.94**	**3.59, 6.80**	***p* < 0.001**	**9.57**	**4.15, 22.07**	***p* < 0.001**	**4.86**	**2.58, 9.17**	***p* < 0.001**	**3.75**	**2.59, 5.65**	***p* < 0.001**
Medicaid expansion	No obs	No obs	No obs	No obs	No obs	No obs	0.90	0.48, 1.70	*p* = 0.755	1.22	0.83, 1.79	*p* = 0.307

Bolded values indicate significance at the < 0.0045 level

OR, odds ratio; CI, confidence interval; No obs, no observation, indicate that all states in the cluster had Medicaid expansion

Covariates in analyses included age, sexual orientation, race/ethnicity, household income, and education level

Cluster 1: California, Connecticut, Maryland, Massachusetts, Minnesota, Montana, Nevada, Oregon, Pennsylvania, and Washington

Cluster 2: Colorado, Washing DC, Delaware, Hawaii, New York, Rhode Island, and Vermont

Cluster 3: Alaska, Arizona, Georgia, Iowa, Maine, Missouri, Nebraska, New Hampshire, New Mexico, Oklahoma, Tennessee, Texas, West Virginia, and Wyoming

Cluster 4: Alabama, Arkansas, Florida, Idaho, Illinois, Indiana, Kansas, Kentucky, Louisiana, Michigan, Mississippi, New Jersey, North Carolina, North Dakota, Ohio, South Carolina, South Dakota, Utah, Virginia, and Wisconsin

## Discussion

This study explored whether lifetime healthcare mistreatment due to gender identity or expression was associated with past-year healthcare avoidance due to anticipated mistreatment among GM people. Approximately 1 out of 3 participants reported having experienced healthcare mistreatment in their lifetime. This is consistent with previous studies’ characterization of the high occurrence of healthcare mistreatment reported by GM people (Glick et al., 2018b; Grant et al., 2011; James et al., 2016).

Approximately 33% of our sample reported past-year healthcare avoidance due to anticipated mistreatment. Despite the growing visibility and acceptance of GM people (Fischer, 2019; Koch-Rein et al., 2020), experiencing healthcare mistreatment in one’s lifetime may continue to impact GM people’s ability and willingness to access healthcare. Reports of lifetime healthcare mistreatment were associated with greater odds of past-year healthcare avoidance due to anticipated mistreatment among all gender identity groups and among groups of GM people who lived in states with similar policies. Healthcare avoidance may result in reduced capability for GM individuals to access important elements of healthcare such as preventative healthcare services (Reisner et al., 2015). Addressing the inadequacy in healthcare providers’ education about transgender health (Lim & Hsu, 2016; Park & Safer, 2018) and by addressing transphobia among already practicing healthcare providers, discrimination may also be reduced, and there may be increased access to care (Poteat et al., 2013; Stroumsa et al., 2019).

The greatest odds of healthcare avoidance among people with a lifetime history of healthcare mistreatment were reported among transfeminine people in our sample. Transfeminine people have widely been found to face mistreatment in healthcare settings and have most frequently been the focus of GM research compared to other subgroups (Cicero et al., 2019). In our sample, the transfeminine subgroup was older compared to the other gender subgroups. This could indicate that transfeminine participants in our sample have a longer history of mistreatment in healthcare that may have informed their decision to avoid healthcare during the past year, which highlights the need for further research elaborating on the nuances in measuring healthcare mistreatment. Supporting GM people to develop coping mechanisms for the fear of mistreatment has been hypothesized as an intervention to increase willingness to access care (White Hughto et al., 2016). Our analysis of models among gender groups (gender-expansive people, transfeminine people, and transmasculine people) adds to a limited body of research examining the experiences of gender-expansive people and reports of healthcare mistreatment and healthcare avoidance due to anticipated mistreatment.

Our study sought to determine whether residing in a state with healthcare policy protections moderated the relationship between lifetime reports of healthcare mistreatment with past-year healthcare avoidance due to anticipated mistreatment. We did not find evidence of moderation among any of the gender groups. We further evaluated the possible differences in the relationship between lifetime healthcare mistreatment and past-year healthcare avoidance among people living in states with similar types of policies, but we did not find discernable differences. We observed this relationship among all clustered groups with the overlapping confidence intervals in each group. However, once the state-level healthcare policy protections variable was added to the model, the effect size of lifetime reports of healthcare mistreatment on past-year healthcare avoidance due to anticipated mistreatment did drop, most dramatically among transfeminine people where it was no longer a statistically significant direct effect. This could speak to some other unaccounted-for characteristics or the need for improved measurement, that may be driving the rates of healthcare avoidance. For example, we cannot discern how policies are implemented in each state, nor can we discern how well policies are enforced once they are passed. Further, we do not know what types of mistreatment occurred, the frequency, or where the mistreatment took place (e.g., emergency room, primary care, inpatient psychiatric care). However, the lack of a statistically significant interaction or direct effect from state-level healthcare policy protections is concerning as we found that participants who reported lifetime healthcare mistreatment were at greater odds of avoiding accessing healthcare during the past year. Ideally, the passage of healthcare policy protections would empower GM people in seeking care, reducing the rate of healthcare avoidance due to anticipated mistreatment, but our findings did not support this relationship. Previous work has found that protective state-level policies were associated with greater use of gender-affirming healthcare services (Goldenberg et al., 2020a), although utilization of other types of healthcare settings is unclear. In our sample, state-level healthcare policy protections did not appear to assuage fears of mistreatment, which influenced GM people’s decision to delay or avoid accessing healthcare services. GM people may lack confidence that the state-level healthcare policy protections would change their personal experiences with healthcare mistreatment. During the time of data collection (June 2017–2018), changes in the sociopolitical environment may have made many GM people feel vulnerable and uncertain of how the changes in the administration would impact their lives (Bockting et al., 2020; Brown & Keller, 2018; National Center for Transgender Equality, 2017). In 2016, parts of the Affordable Care Act, including the provision that protected GM people from healthcare discrimination, were placed on hold due to a Federal district court ruling (Webber, 2016). The federal government had declined to appeal this ruling, signaling a willingness to remove healthcare antidiscrimination protections for GM people entirely (Department Of Health and Human Services, 2019). These publicly discussed and debated changes to the federal policy may have reduced confidence in states’ ability to enforce healthcare policy protections. Since data collection, the federal government has reinstated healthcare antidiscrimination protections for GM people; however, these protections appear to be at the discretion of administrative priorities as well as state-legislative action (National Center for Transgender Equality, 2017; Ronan, 2021). This creates a barrier as GM people have historically had little reason to place trust in institutions as GM people face high rates of incarceration (Graham, 2014; Lambda Legal, 2010), homelessness (Ecker et al., 2019; Eisenberg et al., 2019), among other structural barriers to resources (King et al., 2020). Furthermore, individuals may not understand whether these federal changes will impact their rights and/or experiences in states with healthcare policy protections for GM people. A 2019 study found that almost 50% of GM participants were concerned that rights and protections could be removed imminently; over 40% were not able to correctly identify whether they had protections in healthcare settings in their state, and over 40% believed that there were federal policies in place to allow discrimination against GM people in healthcare (Hughto et al., 2021).

Another consideration is that state-level policies do not necessarily reflect community-level attitudes or societal stigma. For example, there has been a surge of states seeking to prohibit access to gender-affirming care among GM youth and, in the case of Texas, have categorized seeking gender-affirming care as child abuse (Paxton, 2022). However, in Texas, numerous district attorneys in the state have refused to interfere with medical decisions by enforcing the bill (Chudy, 2022) pointing to the nuance between how state-level laws may be passed, but the changes they make in terms of individuals’ experiences may vary based on community-level attitudes. This spectrum of societal stigma in a state creates uncertainty in how policies will be implemented and whether accountability exists should an insurer, facility, or provider violate these state-level policies. This variation between state policy- and community-level societal stigma may impact provider or clinician interactions with GM patients. Some healthcare facilities have implemented changes to address the healthcare needs of GM people (Klein & Nakhai, 2016; Wyckoff, 2019) and in the provision of healthcare education (Cooper et al., 2018; Mayfield et al., 2017; Sherman et al., 2020).

However, little is known about whether these efforts are effective in improving the healthcare experiences of GM people. One study found that transphobia, a type of societal stigma toward GM people, among healthcare providers was a stronger predictor of whether GM patients would experience mistreatment than cultural competency education (Stroumsa et al., 2019). This speaks to how societal stigma in a more localized area, such as a community, may vary in a way that makes the state-level policy a weaker tool for change. Furthermore, communities with greater societal stigma may be slower, or unlikely in general, to incorporate cultural competency interventions in healthcare settings. This speaks to the need for further evaluation of state-level policies with a lens on implementation and systems of accountability for their enactment.

## Limitations

While this study provides insight into how discrimination experiences and healthcare policy relate to anticipated mistreatment due to gender identity or gender expression, there are limitations to our findings. A cross-sectional study design does not allow for the evaluation of a causal relationship; therefore, our findings are strictly correlational. Our sample was predominately White, a higher education level, and of higher socioeconomic status which varies from more representative samples (Badgett et al., 2019). While we did covary these characteristics in our models, the sample composition limits the generalizability of our findings, and replication of these analyses with a representative sample is necessary.

Our measurement of lifetime healthcare mistreatment is limited due to the use of dichotomous response options, resulting in measurement that may not be sensitive enough to capture the change and scope (e.g., frequency, severity) of the experiences of GM people in healthcare settings. Our measurement of past-year healthcare avoidance due to anticipated mistreatment faces similar limitations. We are unable to determine the frequency of healthcare avoidance, the severity of need for the delayed healthcare, nor what types of healthcare were delayed. Additionally, we did not explore the reasons attributed to the reports of past-year healthcare avoidance which could be due to concerns unrelated to gender identity, such as financial concerns or mistreatment related to other marginalized identities one may hold (e.g., race, ethnicity, disability).

We cannot determine how long participants have resided in the state that was reported at the time of survey data collection; therefore, differences based on how recent participants may live in a state with different types of policies are not reflected in our results. Due to the use of multiple policies related to healthcare and the different timing of their passage, we could not evaluate the impact of policies that had been in place for a long time as opposed to states that had only recently passed their policy protections. We also do not evaluate the effect of individual policies that comprised our measure of state-level healthcare policy protections in our models (e.g., “state Medicaid policies related to coverage for transgender people”). These are phenomena that should be evaluated in future work.

Future studies with more diverse samples are needed to provide a detailed analysis of the relationships between healthcare mistreatment, state-level healthcare policy protections, and healthcare avoidance among other groups with intersecting marginalized identities (e.g., racial and ethnic minority groups and sexual minority people). Future studies need to consider how healthcare mistreatment experiences of GM people with other health-related conditions (e.g., disabilities or chronic conditions) may influence healthcare avoidance and use of healthcare services.

Future work to investigate confidence in public institutions and policies could describe the nuanced relationship between policy and behavior, particularly among transfeminine individuals who were found to have the greatest odds of healthcare avoidance in our study. Further, interventions to engage GM people with information on their rights and protections should be explored as opportunities to impact rates of healthcare avoidance. This is especially important because, since the data collection for this study, there has been an unprecedented wave of anti-transgender legislation across much of the USA (Lavietes & Ramos, 2022; Ronan, 2021). As of May 2022 alone, 141 bills that affect GM people were been put forward in 21 states (Freedom for All Americans, 2022). These policies may have a considerable impact on the health and healthcare access of GM people.

## Conclusion

Our study supports the hypothesis that lifetime healthcare mistreatment is associated with healthcare avoidance due to anticipated mistreatment among GM people. However, the presence of state-level healthcare policy protections did not change this relationship. We found that transfeminine people reported the greatest odds of healthcare avoidance and may require additional consideration for support and resources. Sociopolitical changes may have caused confusion and/or impacted the confidence of GM people where state-level healthcare policy protections exist. Further work to identify how state-level policy protections are implemented and the mechanism of accountability is important to contextualize the impact of policy changes. Eliminating mistreatment is critical to increasing access to healthcare to reduce health disparities among GM people.

## Supplementary Information

Below is the link to the electronic supplementary material.

Supplementary file1 (DOCX 28 KB)

## Data Availability

The PRIDE Study is a community-based research project. LGBTQ + populations have historically been marginalized, which has impacted the trust between the communities and institutions. Therefore, part of the guidelines of The PRIDE Study is restricted access to the participants’ data.
