# Supplementary material for: Healthcare Mistreatment, State-Level Policy Protections, and Healthcare Avoidance Among Gender Minority People
Source: Sex Res Social Policy. 2022 Jul 15;19(4):1717–30. doi: 10.1007/s13178-022-00748-1 (PMC9701649; doi:10.1007/s13178-022-00748-1)
Supplement: Supplementary file 1 — Supplementary file1 (DOCX 28 KB) [file 13178_2022_748_MOESM1_ESM.docx]

| Supplemental Table 1. Year of private health insurance non-discrimination policy implementation by state and year of Medicaid expansion if prior to January 2017. | | |
| --- | --- | --- |
| State | Private Health Insurance Non-Discrimination Laws Year Implemented | Medicaid Expansion Year |
| AL | n/a | n/a |
| AK | n/a | 2015 |
| AZ | n/a | 2014 |
| AR | n/a | 2014 |
| CA | 2005 | 2014 |
| CO | 2013 | 2014 |
| CT | n/a | 2014 |
| DC | 2008 | 2014 |
| DE | 2013 | 2014 |
| FL | n/a | n/a |
| GA | n/a | n/a |
| HI | 2017 | 2014 |
| ID | n/a | n/a |
| IL | n/a | 2014 |
| IN | n/a | 2015 |
| IA | n/a | 2014 |
| KS | n/a | n/a |
| KY | n/a | 2014 |
| LA | n/a | 2016 |
| ME | 2014 | n/a |
| MD | n/a | 2014 |
| MA | n/a | 2014 |
| MI | n/a | 2014 |
| MN | 2016 | 2014 |
| MS | n/a | n/a |
| MO | n/a | n/a |
| MT | n/a | n/a |
| NE | n/a | n/a |
| NV | 2011 | 2014 |
| NH | n/a | 2014 |
| NJ | n/a | 2014 |
| NM | n/a | 2014 |
| NY | 2016 | 2014 |
| NC | n/a | n/a |
| ND | n/a | 2014 |
| OH | n/a | 2014 |
| OK | n/a | n/a |
| OR | 2013 | 2014 |
| PA | n/a | 2015 |
| RI | 2016 | 2014 |
| SC | n/a | n/a |
| SD | n/a | n/a |
| TN | n/a | n/a |
| TX | n/a | n/a |
| UT | n/a | n/a |
| VT | 2007 | 2014 |
| VA | n/a | n/a |
| WA | 2014 | 2014 |
| WV | n/a | 2014 |
| WI | n/a | n/a |
| WY | n/a | n/a |
| n/a indicates that the state did not have Medicaid expansion | | |

| Supplemental Table 2. Associations between lifetime healthcare mistreatment and healthcare avoidance due to anticipated mistreatment in transfeminine, transmasculine, and gender expansive groups. | | | | | | | | | | | | |
| --- | --- | --- | --- | --- | --- | --- | --- | --- | --- | --- | --- | --- |
| Predictors | Total Sample | | | Gender Expansive | | | Transfeminine | | | Transmasculine | | |
|  | OR | 95% CI | p | OR | 95% CI | p | OR | 95% CI | p | OR | 95% CI | p |
| Experienced lifetime healthcare discrimination | **4.47** | **3.61, 5.63** | **p<.001** | **4.71** | **3.57, 6.20** | **p<.001** | **10.32** | **4.72, 22.59** | **p<.001** | **3.90** | **2.50, 6.13** | **p<.001** |
| Age | **0.98** | **0.97, 0.99** | **p<.001** | **0.97** | **0.95, 0.99** | **p=.001** | 0.97 | 0.95, 1.00 | p=.083 | 0.99 | 0.97, 1.02 | p=.481 |
| Sexual orientation indicator variables |  |  |  |  |  |  |  |  |  |  |  |  |
| Asexual | 1.15 | 0.88, 1.51 | p=.315 | 1.23 | 0.90, 1.69 | p=.194 | 0.99 | 0.29, 3.33 | p=.984 | 0.84 | 0.40, 1.77 | p=.653 |
| Bisexual | 1.07 | 0.85, 1.34 | p=.565 | 0.91 | 0.69, 1.21 | p=.535 | 2.54 | 1.14, 5.69 | p=.023 | 1.19 | 0.73, 1.93 | p=.493 |
| Gay | 0.82 | 0.62, 1.09 | p=.181 | 0.73 | 0.50, 1.07 | p=.105 | 0.38 | 0.51, 2.74 | p=.334 | 0.94 | 0.55, 1.59 | p=.805 |
| Lesbian | 0.68 | 0.50, 0.93 | p=.014 | 0.71 | 0.48, 1.06 | p=.092 | 0.75 | 0.33, 1.75 | p=.512 | No obs | No obs | No obs |
| Pansexual | 0.96 | 0.76, 1.23 | p=.763 | 1.07 | 0.80, 1.44 | p=.654 | 1.59 | 0.65, 3.88 | p=.312 | 0.65 | 0.37, 1.12 | p=.120 |
| Queer | 1.35 | 1.08, 1.68 | p=.008 | **1.67** | **1.24, 2.26** | **p=.001** | 0.89 | 0.35, 2.28 | p=.812 | 0.79 | 0.50, 1.25 | p=.314 |
| Questioning | **2.20** | **1.33, 3.62** | **p=.002** | 2.19 | 1.09, 4.40 | p=.028 | 3.93 | 1.25, 12.33 | p=.019 | 1.92 | 0.61, 6.07 | p=.267 |
| Same gender loving | 0.80 | 0.52, 1.22 | p=.292 | 0.68 | 0.41, 1.15 | p=.150 | 3.25 | 0.83, 12.83 | p=.092 | 0.70 | 0.24, 2.00 | p=.506 |
| Straight/heterosexual | 1.65 | 1.00, 2.73 | p=.051 | 1.72 | 0.35, 8.43 | p=.502 | 0.75 | 0.13, 4.15 | p=.740 | 1.55 | 0.77, 3.14 | p=.218 |
| Another sexual orientation | 1.17 | 0.79, 1.74 | p=.422 | 1.23 | 0.80, 1.91 | p=.342 | 4.63 | 0.82, 26.08 | p=.082 | 0.41 | 0.56, 3.01 | p=.381 |
| Race and ethnicity indicator variables |  |  |  |  |  |  |  |  |  |  |  |  |
| American Indian/Alaskan Native | 1.14 | 0.68, 1.93 | p=.610 | 1.51 | 0.80, 2.86 | p=.206 | 1.25 | 0.16, 9.94 | p=.832 | 0.55 | 0.15, 2.02 | p=.370 |
| Asian | 0.50 | 0.28, 0.90 | p=.020 | 0.55 | 0.27, 1.12 | p=.099 | 2.13 | 0.27, 16.83 | p=.475 | 0.13 | 0.02, 0.72 | p=.019 |
| Black, African American, African | 0.96 | 0.55, 1.70 | p=.894 | 0.79 | 0.39, 1.63 | p=.531 | 0.30 | 0.12, 6.41 | p=.424 | 1.20 | 0.36, 4.03 | p=.771 |
| Hispanic, Latino, Spanish | 0.89 | 0.55, 1.45 | p=.640 | 0.90 | 0.48, 1.70 | p=.744 | 0.55 | 0.05, 5.73 | p=.619 | 0.91 | 0.35, 2.39 | p=.850 |
| Middle Eastern, North African | 1.27 | 0.56, 2.88 | p=.562 | 1.26 | 0.49, 3.26 | p=.634 | 0.42 | 0.02, 9.60 | p=.586 | 2.63 | 0.27, 25.47 | p=.405 |
| Native Hawaiian, Pacific Islander | 1.61 | 0.44, 5.91 | p=.475 | 2.33 | 0.54, 10.10 | p=.259 | No obs | No obs | No obs | No obs | No obs | No obs |
| White | 0.81 | 0.50, 1.33 | p=.412 | 0.99 | 0.53, 1.87 | p=.979 | 0.29 | 0.04, 7.39 | p=.250 | 0.45 | 0.14, 1.42 | p=.175 |
| Another race or ethnicity | 0.70 | 0.35, 1.40 | p=.312 | 0.52 | 0.22, 1.21 | p=.129 | 0.55 | 0.41, 7.39 | p=.653 | 3.61 | 0.62, 21.00 | p=.153 |
| Socioeconomic covariates |  |  |  |  |  |  |  |  |  |  |  |  |
| Household Income |  |  |  |  |  |  |  |  |  |  |  |  |
| $20,00-$39,999 | 0.95 | 0.68, 1.32 | p=.755 | 1.03 | 0.68, 1.55 | p=.885 | 0.47 | 0.13, 1.67 | p=.244 | 1.23 | 0.59, 2.54 | p=.581 |
| $40,000-$59,000 | 1.01 | 0.71, 1.43 | p=.965 | 1.07 | 0.69, 1.65 | p=.768 | 0.79 | 0.20, 3.09 | p=.730 | 1.00 | 0348, 2.09 | p=.990 |
| $60,000+ | 0.80 | 0.59, 1.10 | p=.165 | 0.93 | 0.64, 1.37 | p=.729 | 1.06 | 0.34, 3.29 | p=.925 | 0.53 | 0.27, 1.05 | p=.069 |
| Education |  |  |  |  |  |  |  |  |  |  |  |  |
| High school, technical school | 1.46 | 0.55, 3.93 | p=.448 | 1.56 | 0.37, 6.54 | p=.543 | 0.19 | 0.01, 4.46 | p=.303 | 1.22 | 0.25, 5.99 | p=.810 |
| College degree (2- or 4-year) | 1.37 | 0.51, 3.67 | p=.537 | 1.26 | 0.30, 5.27 | p=.755 | 0.23 | 0.10, 5.50 | p=.363 | 1.88 | 0.38, 9.32 | p=.439 |
| Graduate degree | 1.01 | 0.37, 2.76 | p=.982 | 1.01 | 0.24, 4.31 | p=.990 | 0.25 | 0.01, 6.39 | p=.523 | 0.89 | 0.17, 4.60 | p=.889 |
| Bolded odds ratios significant at the <.0045 level  OR=odds ratio  CI= confidence interval  Covariates in analyses included age, sexual orientation, race/ethnicity, household income, and education level. | | | | | | | | | | | | |

| Supplemental Table 3. Characteristics of gender minority participants from The PRIDE Study 2018 Annual Questionnaire as grouped in each of the four clusters (N = 2,024) | | | | |
| --- | --- | --- | --- | --- |
| Variable | Cluster 1 | Cluster 2 | Cluster 3 | Cluster 4 |
| n (%) | 877 (43.33) | 225 (11.12) | 321 (15.86) | 601 (29.69) |
| *Personal Characteristics* |  |  |  |  |
| Age, Interquartile Range,  Median | 24.19-37.52, 29.30 | 23.13-34.92,  27.70 | 22.49-33.66,  27.73 | 22.38-33.75,  26.75 |
| Race/Ethnicity (n, %)^a^ |  |  |  |  |
| American Indian or Alaskan Native | 31 (3.53) | 9 (4.00) | 13 (4.05) | 25 (4.16) |
| Asian | 59 (6.73) | 12 (5.33) | 11 (3.43) | 27 (4.49) |
| Black, African American, or African | 27 (3.08) | 12 (5.33) | 9 (2.80) | 24 (3.99) |
| Hispanic, Latino, or Spanish | 45 (5.13) | 15 (6.67) | 25 (7.79) | 18 (3.00) |
| Middle Eastern or North African | 17 (1.94) | 5 (2.22) | 4 (1.25) | 5 (0.83) |
| Native Hawaiian or Pacific Islander | 7 (0.80) | 3 (1.33) | 2 (0.62) | 0 (0) |
| White | 800 (91.22) | 200 (88.89) | 299 (93.15) | 567 (94.34) |
| Another race/ethnicity than is listed | 25 (2.85) | 6 (2.67) | 6 (1.87) | 10 (1.66) |
| Sexual Orientation^a^ |  |  |  |  |
| Asexual | 157 (17.90) | 34 (15.11) | 54 (16.82) | 108 (17.97) |
| Bisexual | 260 (29.65) | 70 (31.11) | 97 (30.22) | 199 (33.11) |
| Gay | 152 (17.33) | 50 (22.22) | 60 (18.69) | 106 (17.64) |
| Lesbian | 163 (18.59) | 35 (15.56) | 52 (16.20) | 89 (14.81) |
| Pansexual | 208 (23.72) | 45 (20.00) | 90 (28.04) | 157 (26.12) |
| Queer | 540 (61.57) | 148 (65.78) | 174 (54.21) | 326 (54.24) |
| Questioning | 26 (2.96) | 11 (4.89) | 16 (4.98) | 28 (4.66) |
| Same-Gender Loving | 71 (8.10) | 13 (5.78) | 27 (8.41) | 35 (5.82) |
| Straight/Heterosexual | 38 (4.33) | 12 (5.33) | 20 (6.23) | 22 (3.66) |
| Another Sexual Orientation | 70 (7.98) | 15 (6.67) | 15 (4.67) | 41 (6.82) |
| Gender Identity |  |  |  |  |
| Gender Expansive | 583 (66.48) | 143 (63.56) | 177 (55.14) | 387 (64.39) |
| Transfeminine | 110 (12.54) | 33 (14.67) | 46 (14.33) | 74 (12.31) |
| Transmasculine | 184 (20.98) | 49 (21.78) | 98 (30.53) | 140 (23.29) |
| *Socioeconomic Characteristics* |  |  |  |  |
| Education Level |  |  |  |  |
| No High School Degree | 9 (1.03) | 1 (0.44) | 6 (1.87) | 8 (1.33) |
| High School/GED Graduate or Some College | 265 (30.25) | 68 (30.22) | 132 (41.12) | 229 (38.17) |
| College Degree, 2- or 4-year | 355 (40.53) | 88 (39.11) | 122 (38.01) | 228 (38.00) |
| Graduate Degree | 247 (28.20) | 68 (30.22) | 61 (19.00) | 135 (22.50) |
| Household Income |  |  |  |  |
| <$20,000 | 118 (13.75) | 32 (14.75) | 48 (15.24) | 90 (15.36) |
| $20,000-$39,999 | 161 (18.76) | 48 (22.12) | 77 (24.44) | 172 (29.35) |
| $40,000-$59,999 | 158 (18.41) | 35 (16.13) | 68 (21.59) | 98 (16.72) |
| $60,000+ | 421 (49.07) | 102 (47.00) | 122 (38.73) | 226 (38.57) |
|  |  |  |  |  |
| Experienced lifetime healthcare mistreatment | 304 (34.66) | 63 (28.00) | 98 (30.53) | 219 (36.4) |
| Avoided healthcare due to fear of mistreatment within the past year | 313 (35.69) | 83 (36.89) | 103 (32.09) | 177 (29.45) |
| ^a^variables were not mutually exclusive; therefore, totals may add up to over 100% | | | | |
